# Therapeutic Effects of Nano-HAp in a Rat Model of AlCl_3_ Induced Neurotoxicity

**DOI:** 10.22037/ijpr.2019.1100760

**Published:** 2019

**Authors:** Osma Ahmed Abbas, Issa Ghada Ibrahim, Abdel-Gawad Eman Ismail

**Affiliations:** *Radioisotopes Department, Egyptian Atomic Energy Authority, Cairo, Egypt.*

**Keywords:** Nano-hydroxyapatite, Aluminum chloride, Nuclear respiratory factor1, Fragmented DNA, Caspase3

## Abstract

With the advance in nanomedicine, the present study was conducted to explore the possible therapeutic role of intravenous nano- hydroxyapatite (nano-HAp) in male rats after chronic exposure to aluminum chloride (AlCl_3_). This exposure interposed DNA fragmentation, apoptosis, alters oxidant/antioxidant status as well as change in content of neurotransmitters. The rats were injected with 100 mg/kg. body weight (b.w.) of AlCl_3_ intraperitoneally for 90 days, after then nano-HAp was injected intravenously (i.v.) three times per week at a dose level 100 mg/kg b.w. Based on the results obtained, it can be concluded that the treatment with the prepared nano-HAp restrains the damage inflicted on brain modulation by lipid oxidation products and decreased the susceptibility of apoptotic cells death with subsequent repaired the fragmented DNA as well as improved the synthesis of neurotransmitters. The most salient finding of nano-HAp treatment is the disappearance of most pathological changes due to AlCl_3_ administration.

## Introduction

Aluminum (Al) is ubiquitous element in the environment and released either naturally through weathering and erosion processes or from various anthropogenic sources. Although this element has a myriad of uses in daily life and many of its salts are used in the pharmaceutical industry and in treating drinking water as flocculants, yet it is a neurotoxic metal that may be involved in the progression of neurodegenerative changes (1).The emergence of extensive evidence demonstrated the adverse effects of aluminum in inducing memory impairment, personality changes, and dementia in humans. It activates neurotoxicity in central nervous, skeletal, and hematopoietic systems. Aluminium has the ability to produce neurotoxicity by many mechanisms. Beside, promotion of insoluble beta-amyloid (A beta) and hyperphosphorylated tau protein formation and accumulation, Al can alter neuronal signal transduction pathways associated with glutamate receptors (2). It has been suggested that it interferes with glutamatergic neurotransmission and impairs hippocampal long-term potentiation by disrupting the glutamate- Nitric oxide -cyclic guanosine monophosphate signaling pathway. On the other hand, stress associated with Al exposure induced change in the catecholamine levels (3). Catecholamine, including dopamine and norepinephrine, are the principal neurotransmitters that mediate a variety of the central nervous system function and are involved in different neurodegenerative disorders. The main sites of catecholamines production are brain, chromaffin cells, and the sympathetic neurons (4).

Nowadays, reactive nanoparticles (NPs) as energetic materials have received much recent attention for a variety of existing and/or potential applications. Because the NPsare of comparable length scale as discrete components and proteins that make up a cell, they may potentially evade the cellular defense mechanism leading to permanent cell injury. Consequently, the interaction of NPs with biological systems such as living cells has become one of the most fascinating areas of science and applied research that lies at the interface of nanotechnology and biology. Nano-hydroxyapatite (nano-HAp) is among the many nano materials (NMs) that are intentionally added into oral hygiene products. One of the thermodynamic advantages of nano-HAp, is the most stable phase of it in physiological conditions. Nano-HAp has a good biodegradability in situ, high biocompatibility, and excellent osteoconductive as well as osteoinductive capabilities depending on its composition, structure, morphology, and crystallite size (5). Also, the chemical similarity of this material with bone and teeth as well as its excellent biocompatibility and bioactivity has attracted the attention of medical professionals. The extensive research on biological and physio-chemical properties of this material has widened its scope of application. In recent years, it has found promising application in other areas of medicine due to the similar dimensions to the inorganic components of calcified tissues, higher specific roughness surface area, and strong interaction with organic materials compared to conventional HAp materials (5, 6). Since the early studies on this subject, up to now, the most important biological end point has been the various uses of HAp in medical field especially hepatic tumor (7). It has been frequently used as genetic carrier (8), drug delivery (7), repairing material of bone defect in clinic (9, 10). Recently, some studies showed that nano-HAp have significant effect in removal of lead toxicity of the body (11), particularly the liver (12). Some studies reported that HAp nanoparticles have biotoxicity which is affected by the diameter of the particles, exposure dose, and contact way (13). In addition, the concentration of nano-HAp has significant effect on the biological response (14, 15). Therefore, cautions should be exercised before using these nanoparticles as the size and morphology.

Based on the hypothesis that nano-HAp may be effective in the management of heavy metal-induced disease and the progress of fabrication nano-HAp with improved biological properties evidently, the current study was conducted to evaluate the performance of a prepared nano-HAp in overcoming brain damage induced by AlCl_3_ in rats. Biochemical and pathological brain investigations were performed to evaluate the effectiveness of the prepared of nanoparticles.

## Experimental

*Chemicals*

The chemicals used for HAp preparation were calcium nitrate tetrahydrate (Ca (NO_3_)_2_.4H_2_O, with molecular weight (Mwt) 236.15 g/mole, Merk, Germany), diammonium hydrogen orthophosphate anhydrous ((NH_4_)_2_HPO_4_, Mwt 132.06g/mole, S.D. Fine Chem. Ltd. Mumbai, India), and ammonium hydroxide (NH_4_OH, Mwt. 35.5g/mole, May & Baker, England). Aluminum chloride hexahydrate (AlCl_3_. 6H_2_O) was purchased from Sigma-Aldrich.

All chemicals and reagents used were of analytical grade and used without further purification.

*Preparation of nano-HAp*

The nano-HAp prepared according to (16) by preparing reacting of 0.497M/L of Ca(NO_3_)

.4H_2_O and 0.298M/L of(NH_4_)_2_HPO_4_which were dissolved separately under pH control. The obtained precipitates were separated from the mother liquor by filtration and dried at 90 °C and calcined at 1000 °C for 24 h. The infrared spectra of the products were carried out with Mattson Infinity Series FTIR made in USA, in the wave number range from 400-4000 cm^-1^ using the KBr disc technique. The samples were characterized for qualitative and quantitative phase content by X-ray diffraction (XRD) by using Shimadzu X-ray diffractometer made in Japan. An XRD analysis was performed after calcining the synthesized powder to reveal the structural of the prepared powder after heat treatment.

*Pharmacological Study of Acute Toxicity*

Determination of acute toxicity for intravenous treatment with nano-HAp was carried out using the method of previously published by Lorke (17). Fifteen rats were used to determine the toxicity of the prepared nano-HAp. The rats were divided into five groups of three rats each. The rats were injected intravenously with nano-HAp at dose levels of 100, 200, 300, 500, 1000 mg/kg b. w. Mortality was recorded for near 24 h and the final LD_50_ value was determined from the minimum concentration (full death) and maximum concentration (no death) of the dose according to the coming relation:

LD_50_ = (M_0_+ M_1_) /2

Where M_0_ = Highest dose of nano-HAp that gave no mortality, M_1_ = Lowest dose of nano-HAp that gave mortality.

*Preliminary study using nano-HAp to treat brain damage*

Several experiments were carried out to evaluate the pathophysiological features of brain in rats intoxicated by AlCl_3_ before and after treatment with nano-HAp. The selected dose of nano-HAp was examined by the intravenous injection at different time intervals to detect the optimal therapeutic results. 

It was reported that after i.v. injections of 100 mg/kg b.w. nano-HAp (12), the particles stay in the blood until they enter the reticuloendothelial system (macrophages of the liver, spleen and bone marrow). The LD_50_ of this preparation was determined as 1200 mg/kg b.w. (15). Plasma half-life time of one hour has been reported by Xie (18). However, structural and functional changes in brain were investigated including DNA fragmentation, neurotransmitters, and oxidation status. A divided dose of 300 mg/kg b.w. nano-HAp (a quarter dose of LD_50_) was applied in the present experiment.

*Animals and treatment schedule*

Twenty-four male albino rats were kept under hygienic conditions with a 12/12 h light - dark cycle and were allowed free access to food and water *adlibitum *for at least one week prior to the experiment.

The animals were assigned into three groups (n=8). Group 1: control rats. Group 2: rats administered AlCl_3_ (100 mg/kg b.w.) intraperitoneally for 90 days. Group 3: rats injected intravenously with nano-HAp (dissolved in saline, 100 mg/Kg b.w.) three times/week (11, 12) after being induced by AlCl_3_ for 90 days, for one week. The blood samples were collected from orbital venous plexus of all rats 24 h post last injection of nano-HAp for biochemical estimation. The brain tissues were obtained from the animals for DNA fragmentation and histological examination. 

*Tissue Preparation*

At the end of treatment period, the rats were anaesthetized by diethyl ether and sacrificed by cervical decapitation. The brains were dissected out by making midline incision to view the skull. A small incision from the caudal part of parietal bone and a firm cut in the anterior part of the frontal bone were made to remove the brain more easily. The isolated brains tissues were immediately taken out and washed with ice cold saline to remove blood and they were either fixed in 10% formalin for histopathological examination or stored at -80 °C, till later analysis.

**Figure 1:**
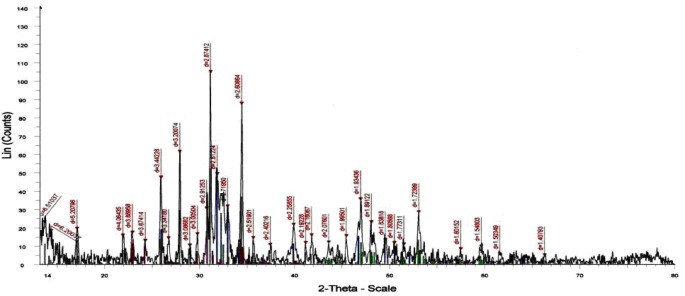
XRD patterns of the sample prepared with Ca/P molar ratio 1.67 under pH control calcined at 1000 °C

**Figure 2:**
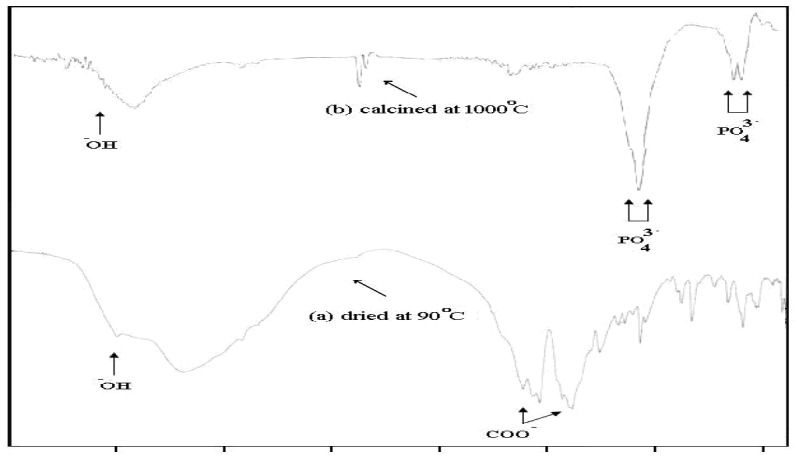
IR spectra of the sample prepared with Ca/P molar ratio 1.67 under pH control dried at 90  C and which calcined at 1000 °C

**Figure 3:**
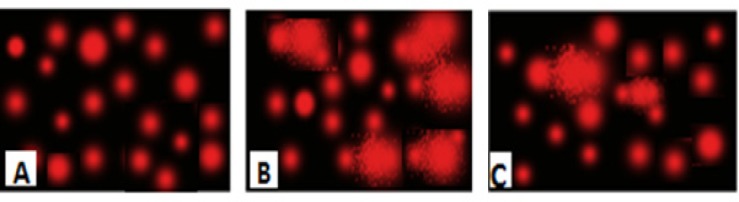
Photomicrograph represented DNA damage (Comet assay) in brain of normal rats (A), rats administered AlCl3 for 90 days(B) and rats treated with nano-HAp (C).

**Figure 4:**
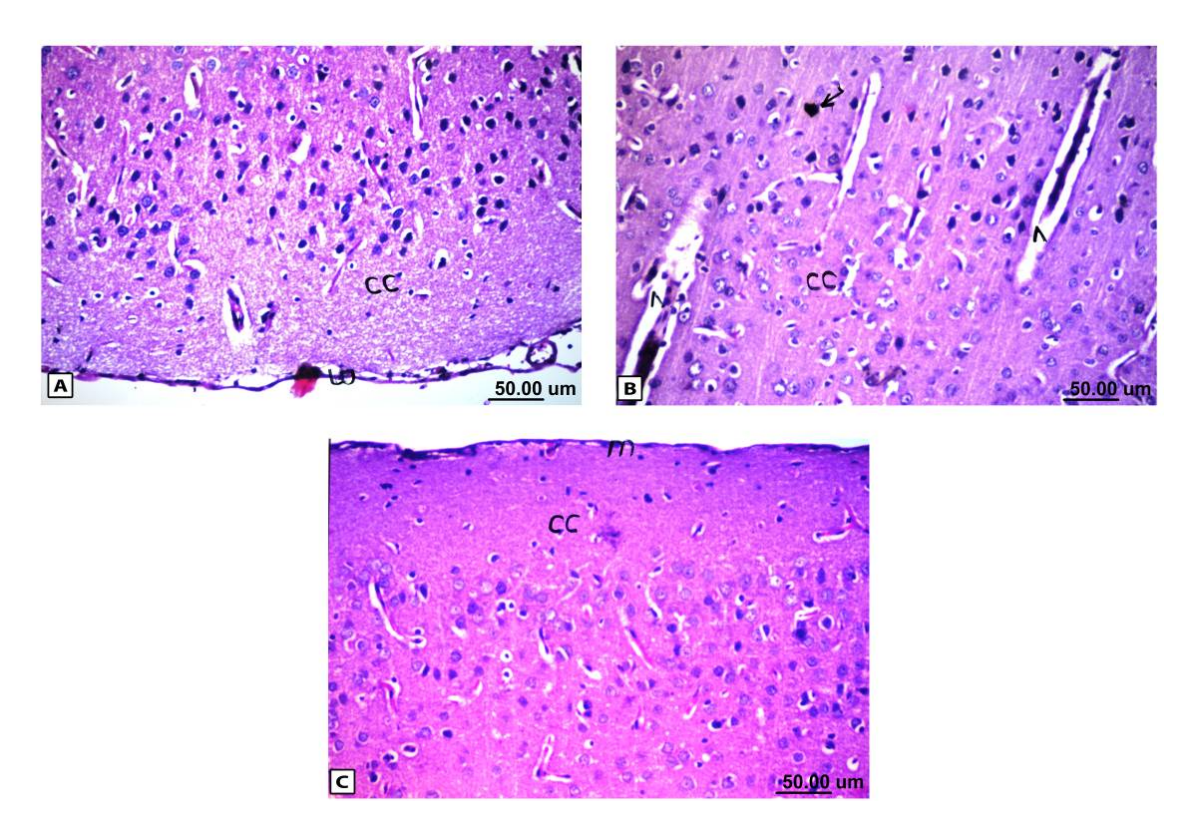
Histopathological results of rat brain area of meninges (m) and cerebral cortex (cc). (A) Normal control group: normal structure of meninges (m) and cerebral cortex (cc). (B) Rat administered AlCl3: the cerebral cortex showed congestion in the blood capillaries with degeneration in some neuronal cells. (C) Rat treated with i.v. nano-HAp after AlCl3: normal structure of meninges (m) and cerebral cortex (cc)

**Figure 5:**
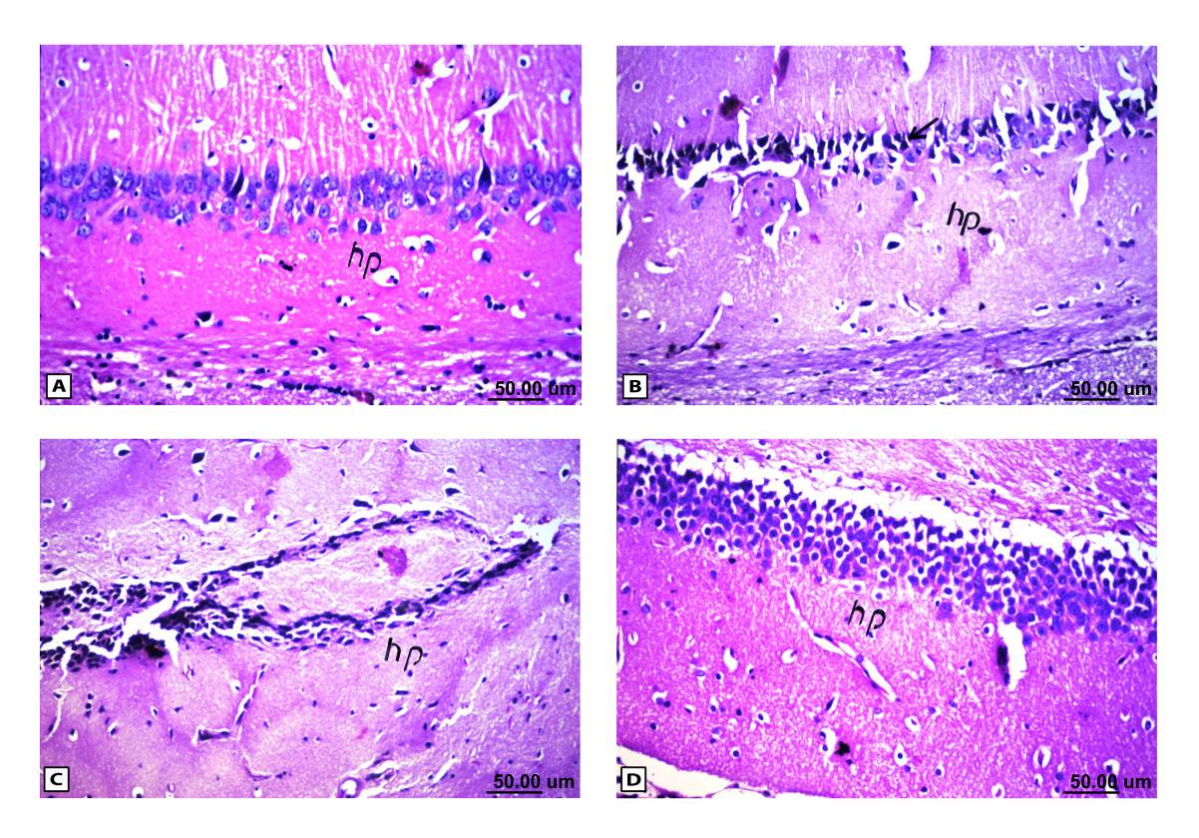
Histopathological results of rat brain area hippocampus (hp). (A) Normal control group: normal structure of the hippocampus (hp). (B & C) Rat administered AlCl3: neuronal degeneration and pyknosis (arrow) in hippocampus (hp) & atrophy hippocampus. (D) Rat treated with i.v. nano-HAp after AlCl3: normal structure of the hippocampus (hp)

**Figure 6:**
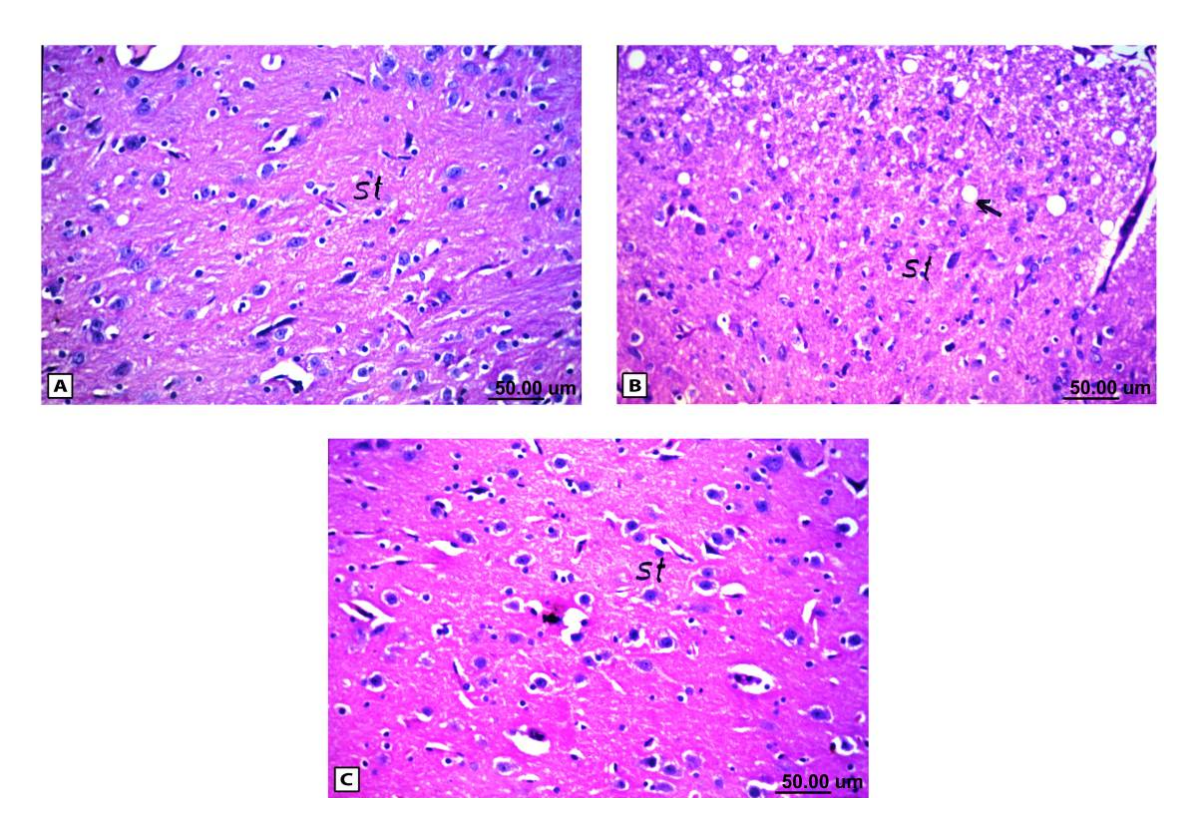
Histopathological results of rat brain area of striatum (st). (A) Normal control group: normal structure of striatum (st). (B) Rat administered AlCl3: vacuolization in the matrix of striatum (arrow). (C) Rat treated with i.v. nano-HAp after AlCl3: Normal structure of striatum (st)

**Figure 7:**
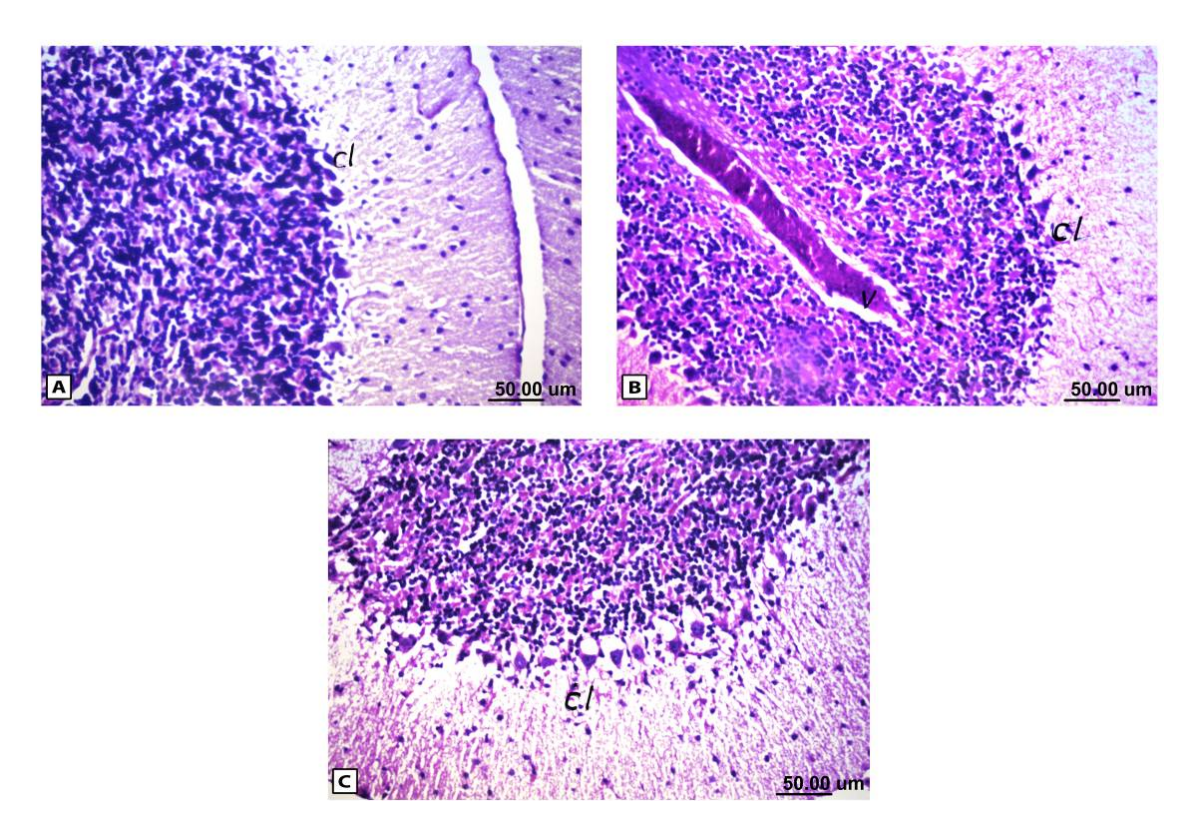
Histopathological results of rat brain area of cerebellum (cl). (A) Normal control group: normal structure of cerebellum (cl).

(B) Rat administered AlCl3: Congestion in blood vessels (v) in cerebellum (cl). (C) Rat treated with i.v. nano-HAp after AlCl3: normal structure of cerebellum (cl).

**Figure 8:**
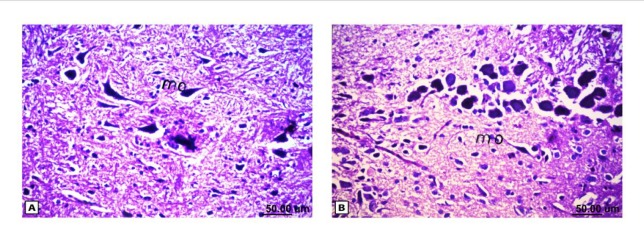
Histopathological results of rat brain area of medulla oblongata. (A) Normal control group: normal medulla oblongata structure. (B) Rat treated with i.v. nano-HAp after AlCl3: normal medulla oblongata structure

**Table 1:** IR analysis of samples dried at 90 °C and calcined at 1000 °C

**Type of spectrum**	**Vibration of hydroxyl ** **-** **OH υ (cm** **-1** **)**		**Phosphate PO ** **3-** **stretching** **4** **vibration υ (cm** **-1** **)**			**Phosphate PO ** **3- ** **bending** **4** **vibration υ (cm** **-1** **)**	
HAp bands	630	3570	963	1036	1091	565	603
Sample dried at 100°C	-	3570	980	1054	1079	539	600
Sample calcined at 1000°C	630	3567	-	broad	1180	569	603

**Table 2:** Score of DNA damage in cells from different groups of male rat

	**Tailed %**	**Untailed %**	**Tail length (µm)**	**Tail DNA %**	**Tail moment UNIT**
Control	3	97	1.22±0.04	1.31	1.59±0.05
AlCl3	21	79	3.81±0.24	3.96	15.05±1.08
AlCl3+nano-HAp	16	84	2.89±0.11	2.93	8.47±1.01

**Table 3:** Effect of nano-HAp on neurotransmitters in different groups

Groups	**Control**	**AlCl** **3**	**Nano-HAp+AlCl** **3**
**Parameters **			
5-HT(ng/100mg)	8.56±0.17a	6.02±0.12b	7.90±0.22a
NA (ng/100mg)	6.92±0.13a	4.56±0.14b	6.22±0.15c
Dopamine (ng/100mg)	25.99±0.34a	17.82±0.38b	28.09±0.81a

Values represent means ± S.E.

Values bearing different superscript in the same raw are statistically different.

**Table 4:** Effect of nano-HAp on sphingomyelin, NRF1and caspase-3 in different groups

Groups	**Control**	**AlCl** **3**	**Nano-HAp+AlCl** **3**
Parameters			
Sphingomyelin(mg/gwet)	2.83±0.07a	1.94±0.03b	2.82±0.10a
NRF1(ng/100mg)	24.96±0.98a	11.77±0.36b	16.64±0.42c
Caspase3 (ng/100mg)	111.95±0.72a	168.04±1.66b	137.23±1.69c

Values represent means ± S.E.

Values bearing different superscript in the same raw are statistically different.

**Table 5:** Effect of nano-HAp on GPx and MDA in different groups

Groups	**Control**	**AlCl** **3**	**Nano-HAp+AlCl** **3**
Parameters			
MDA (nmol/mg)	0.96±0.07a	4.27 ±0.10b	2.04 ±0.10c
GPx (nmol/mg)	37.06 ±0.73a	19.83 ±0.54b	30.67 ± 1.60c

Values represent means ± S.E.

Values bearing different superscript in the same raw are statistically different.

**Table 6:** Severity of the reaction in brain of different groups according to the histopathological alterations

**Control**	**AlCl3**	**AlCl3+Nano-HAp**
-	++++	-

Very sever ++++, sever +++, moderate ++, mild +, nil -

*DNA fragmentation*

Brain DNA damage was determined by a single-cell gel electrophoresis (comet) assay according to the method previously published by Singh *et al.*, (19). A 0.5 g of crushed brain sample was transferred to 1 mL ice-cold phosphate buffer saline (PBS). The suspension was stirred for 5 min then filtered. Cell suspension (100 µL) was mixed with 600 µL of low-melting agarose (0.8% in PBS). 100 µL of this mixture was spread on pre-coated slides, which were immersed in lyses buffer (0.045 M TBE, pH 8.4, containing 2.5% SDS) for 15 min. The slides were placed in electrophoresis chamber containing the same TBE buffer, but devoid of SDS. The electrophoresis conditions were 2 V/cm and 100 mA for 2 min. Staining was made with Ethidium bromide (EtBr) 20 µg/mL at 4 °C. The observation was reported while the samples still humid, the DNA fragment migration patterns of 100 cells for each dose level were evaluated with a fluorescence microscope (With excitation filter 420-490 nm (issue 510 nm). For visualization of DNA damage, observations were made of EtBr-stained DNA using a 400X objective on a fluorescent microscope. The comets tails lengths were measured from the middle of the nucleus to the end of the tail.

*Comet capture and analysis*

A total of 100 randomly captured comets from each slide were examined at 400 X magnification using a fluorescence microscope connected to a CCD camera using an image analysis system [Comet 5 image analysis software developed by Kinetic Imaging Ltd. Liverpool, UK]. A computerized image analysis system acquires images, computes the integrated intensity profiles for each cell, estimates the comet cell components, and then evaluates the range of derived parameters. To quantify the DNA damage, the tail length (TL), the percentage of migrated DNA (tail DNA %), and tail moment (TM) were evaluated. TL (length of DNA migration) is related directly to the DNA fragment size and presented in micrometers. It was calculated from the center of the cell. Finally, the program calculates TM.

The DNA damage was quantified by measuring the displacement between the genetic material of the nucleus (Comet head) and the resulting (tail). 

$\mathrm{Tail DNA\%}=\frac{\mathrm{Tail DNA intensity}}{\mathrm{Cell DNA intensity}}\times 100$

Tail moment = Tail DNA % X Length of tail

*Biochemical Estimation*

The obtained tissue was analysed for the following: sphingomyelin, using a commercial quantification colorimetric assay kit purchased from BioVision (CA, USA. Catalog # K600-100), nuclear respiratory factor 1 (NRF1) and caspase-3 of brain were measured using ELISA commercial kits purchased from USCN Life Science Inc., (Wuhan, China. number SEC669 Ra and SEA626 Ra. respectively). Neurotransmitters such as catecholamine (dopamine and noradrenalin) and 5-hydroxytryptamine (5-HT) were also evaluated according to the method reported by Zagrodzka *et al*. (20). Lipid peroxidation products were determined by measuring the quantity of malondialdehyde (MDA) produced using colorimetric/fluorometric kit purchased from (BioVesion Research Products, USA, Catalog number K739-100). The enzyme activity of Glutathione peroxidase (GPx) was assayed using kinetic kit purchased from (BioVesion Research Products, USA, Catalog number K762-100)

*Histopathological examination*

For histopathological examination through the electric light microscope, the samples were taken from brain of eight rats in each group and fixed in 10% formal saline for twenty four hours then washed with tap water. Serial dilutions of alcohol (methyl, ethyl and absolute ethyl) were used for dehydration. Specimens were cleared in xylene and embedded in paraffin at 56 ⁰C in hot air oven for twenty four hours. Paraffin bees wax tissue blocks were prepared for sectioning at 4 microns by sledge microtome. The obtained tissue sections were collected on glass slides, de-paraffinzed, and stained by hematoxylin and eosin stains (21)

*Statistical analyses*

All values were expressed as mean ± S.E. Statistical analysis was performed with one way analysis of variance (ANOVA) followed by Duncan’s test using SPSS program. *Ρ* values < 0.05 were considered to be statistically significant.

## Results

*Characterization of the prepared nano- HAp*

The XRD analysis of the dried powder calcinated at 1000°C identified the presence of HAp crystal structure (15) without any other calcium phosphate structures as shown in Figure (1). The FTIR analyses of the prepared nano-HAp dried at 100 °C and that calcined at 1000 °C are shown in Figure 2 and Table (1). The two bands at 630 and 3570 cm^-1^ belonged to the vibration of hydroxyl OH gradually appeared. Those bands at 1036, 1091, and 963 cm^-1^ are characteristic for phosphate (PO4^3-^) stretching vibration, while the bands at 603 and 565 cm^-1 ^were due to phosphate bending vibration (15).

*DNA fragmentation*

An increase in DNA damage was detected by the comet assay and was indicated by an increase in migration length of the stained DNA as shown in Table 2. The extent of DNA damage, measured in TM, increased after administration of AlCl_3_ when compared with corresponding controls. Following single-cell electrophoresis, the lengths of the comets (DNA tails) depended on the effect of AlCl_3_ stress on rats. The longer tails indicating more DNA damage. Figure 3 represented the effect of treatment with nano-HAp on the damage DNA, induced by AlCl_3_. Control brain rat DNA showed no tails (Figure 3A). The tails were detected after AlCl_3_ administration (Figure 3B). The mean value of tail length in the AlCl_3_ group increased rapidly and significantly compared with their controls, and more DNA was observed in the tail (Figure 3). The extent of damage decreased significantly in the nano-HAp treated group to a great extent as shown in (Figure 3). However, it is worth mentioning that it could not be comparable to the control.

*Biochemical results*

As shown in table 3, the rats administered AlCl_3_ showed significant decrease (*p* < 0.05) in the neurotransmitters concentration; 5-HT and catecholamine (noradrenalin (NA) and dopamine) in brain tissue, treatment with the nano-HAp, that followed AlCl_3 _administration restored the levels of these neurotransmitters in the treated group. 

Table (4) showed that exposure to AlCl_3 _induced a significant decrease in brain concentration of sphingomyelin and NRF1 but the level of caspase3 showed significant augmentation as compared to the control animals. On the other hand, compared to group received AlCl_3_ only, the intravenous nano-HAp restored sphingomyelin level besides modulating the levels of NRF1 and caspase3 to a great extent. Table 5 showed the activity of MDA and GPx enzyme in brain tissue. Exposure to AlCl_3 _is detrimental factor to the redox status as evidenced by a significant rise (*P*< 0.05) in MDA level and significant depletion (*P*< 0.05) in GPx activity related to the control. As compared to the group administered AlCl_3_, the group treated with the nano-HAp showed significant increases in the activity of GPx and decreases in the level of MDA.

*Histopathological observations*

As shown in Figure (4), the control brain sections exhibited typical structure of the meninges and cerebral cortex (Figure. 4A), hippocampus (Figure.5A), striatum (Figure 6A), cerebellum (Figure.7A) and medulla oblongata (Figure 8A). On the other hand, brain section of rats administered AlCl_3_ showed congestion in the blood capillaries with the degeneration in some few neuronal cells in cerebral cortex (Figure 4B). In addition, degeneration and pyknosis was observed in the neuron of the atrophied hippocampus (Figures 5B, C). There was vacuolization in the matrix of striatum (Figure 6B) and congestion in the blood vessels of the cerebellum (Figure 7B). As displayed by the composite nano-HAp, the sections of brain collected from rats treated with the nano-HAp showed restore of histological structure of all parts of brain, meninges, cerebral cortex (Figure.4C), hippocampus (Figure 5D), striatum (Figure 6C), cerebellum (Figure 7C),and medulla oblongata (Figure 8B).The severities of histopathological alterations were summarized in table 6.

## Discussion

Hydroxyapatite has been widely used as a biomaterial for oral cavity medicine and has good issue compatibility both outside and inside the body (22). Because nano-HAPs cause very small excitation of the blood vessels, feeding medicine could be made by intravenous injection (23). In this study, the nano-HAp was synthesized by organic-inorganic reaction in polymeric matrix rout and showed a good dispersive effect, and besides the very uniform size (50 nm), it has advantage of high surface energy. The prepared nano-HAp was examined as therapeutic drug for repairing the function and structure of brain cells after chronic exposure to AlCl_3_. The forgoing literatures reported the high capacity of the nano-HAp in the removal of heavy-metal ions in-vitro and in-vivo. But researches-conducted on the efficiency of this nanoparticles to overcome the brain injury especially, after exposure to accumulation of heavy metals were nearly absent (24, 25, 11). The only known study was carried out on radiosensitization of tumor cells induced by the nano-HAp in mice bearing breast tumor brain (26). Thus, any comparison with the literature is difficult. However, it is possible to rely on the mode of action of nano-HAp that has been proved in other cases.

The present study showed that chronic administration of AlCl_3_ could lead to apoptosis as seen in the micrographs which clearly revealed disruption of cells, increased fragmentation of DNA and the number of comets were observed. It is commonly known that the usage of comet assay allowed the detection of DNA alterations of the diverse types, such as double-strand breaks, single-strand breaks, alkali-labile sites, incomplete repair sites, and cross-links (27). Damage to DNA is one of the markers and typical characteristic of apoptosis and the percentage of DNA in the tail is the most appropriate parameter to analyze induced DNA damage (2). Several lines of evidence indicated that the excessive production of reactive oxygen species (ROS) resulted from exposure to AlCl_3_ leads to DNA oxidative damage (1, 28, 29), where the hydroxyl radicals remove hydrogen from nucleic acids or react with double bond (28). Moreover, Al is considered as a stress-inducing agent in endoplasmic reticulum and potentially opens the mitochondrial permeability transition pore, which in turn can further stimulate ROS production, worsen energy failure and activate the expression of various genes that are important in growth arrest and DNA damage induction (30). Among these genes is NFR1 which is essential for the integration of nuclear- and mitochondrial encoded gene transcription; coordinates nuclear DNA synthesis and mitochondrial function (31)*.* NRF1 expression is stimulated by endogenous physiological events and bind to the antioxidant response element thereby mediating the transcription of anti-oxidative metabolizing enzymes (32).

Al as known has a fixed oxidation number and, therefore hardly participate in redox reaction but induce oxidative damage on neural cells indirectly by increasing the redox active iron concentration in the brain mainly via the Fenton reaction (33, 34). The performance of AlCl_3_ in aggravating oxidation was manifested in the present study by significant increase in brain MDA accompanied by concomitant decrease in the activity of GPx in brain tissue. Thus, they have serious bearing on the functional and structural development of the central nervous system such as reduction of axonal mitochondria turnover, disruption of Golgi, and reduction of synaptic vesicles (35). The relationship between oxidative stress and neuronal death has been extensively investigated (30, 36). It was reported that oxidative stress release pro-apoptotic factors into the cytoplasm *via *activation of the Jun amino-terminal kinases (JNK) pathway or by activation of nuclear factor (NF-κB) and transcription factors accompanied by marked inhibition of anti-apoptotic protein like Bcl-2 (37). JNK is important for Aβ induction of neuronal death mediated by caspase3 (38). During apoptosis, a specific nuclease (caspase-activated family, CAD), cuts the genomic DNA between nucleosomes which generates apoptotic chromatin condensation and DNA fragments. Caspase-3 is frequently activated death protease, catalyzing the specific cleavage of many key cellular proteins (30). Caspas-3 acts as a new pro-caspase-3 synthesis and formation of other proteins required for caspase-3 activation (39).

It has also been suggested that the lipid oxidation milieu of long exposure to AlCl_3_ leads to reduction in content of brain sphingomyelin which is considered as a major constituent of lipid myelin sheath in nerve cell membranes and have been also identified as major lipid components in membrane rafts (40). In addition, the choline required for neurotransmitter synthesis, it may be extracted from sphingomyelin (41). According to this hypothesis, in order to make up for the choline deficiency, the neurons try to extract choline from sphingomyelin components and this led to the disruption of cell membranes and ultimately to neuronal cell death (41).

Catecholamine, including dopamine and norepinephrine, are the principal neurotransmitters, synthesized in the brain and mediate a variety of the central nervous system functions (42).The neurotoxicity of Al is directly linked to its bioavailability. Serum Al binds to the transferrin (an iron carrier protein) and will be taken up by the especial receptor in the cell membrane of brain capillaries and can eventually mediate its absorption into the brain (43). Another possible mechanism for the catecholamine reduction is related to the competition of Al with either Mg and/or Zn. These elements may be involved in the conversion of dihydroneopterin triphosphate to tetrahydrobiopterin. This product is responsible for the production and also regulation of catecholamine synthesis (3). Therefore, the reduction in the catecholamine content may, however, be as a result of either decreasing in the catecholamine production following Al accumulation in brain or might be related to the preventive role of Al to release this neurotransmitter (44).When the action potential reaches the presynaptic membrane, it was reported that it alters the membrane polarization that allowed the entrance of calcium ions, resulting in vesicular fusion and releasing noradrenalin. It is pertinent to mention that the direct precursor of noradrenalin is dopamine, occurring predominantly in the neurotransmitter vesicle (42). On the other hand, 5-hydroxytryptamine (5-HT) is involved in neuroendocrine functions and the balance of physiological activities of brain could be altered by replacing Al the metal ions in many enzyme systems (39). In this aspect, it was reported that Al promoted decreases in 5-HT level after 60 days treatment due to disruption of tryptophan metabolism. This restraining effect of aluminum salt on the enzymes managed the synthesis and correlation of neurotransmitters, manifested in the present study through the reduction of neurotransmitters content, which is dopamine, noradrenalin and 5- HT in brain.

The obtained data demonstrated, for the first time, that the nano-HAp succeeded to a great extent in recovery of the functional alteration associated with neurotoxicity of AlCl_3_ and these findings were evidenced in modulating the oxidant/antioxidant status represented in MDA, GPx, and sphingomyelin with subsequent repair of fragmented DNA and the decreased susceptibility to apoptotic cell death as well as improvement of neurotransmitters. Thus, the nano-HAp potentiated redox homeostasis status and has challenging role in quenching free radicals. Previously, it was attributed the antioxidant effect of the nano-HAp to the scavenging of superoxide (11, 45), which is the main component of oxidative stress or to inhibition of oxidative and nitro-oxidative species formation (46). Because the oxidative stress is the manager for internal and/or external factors induced adverse effect, the decreased DNA damage and the diminished activation of caspase-3 observed in the treated group were the expected results (12) which considered great indicators for reduction of neurodegeneration upon the treatment with nano-HAp.

Another important consideration is nano particles developed and can cross the blood brain barrier due to their extremely small size. Additional impediments are of the biological barrier type, which includes the reticular endothelial system (RES), consisting of phagocytes. On the other hand, nano–HAp prepared from chitosan which has a variety of ligands specific for cell surface receptors to increase recognition and uptake of nano- material into cells through receptor-mediated endocytosis (47). In addition, the increase of permeability of the blood brain barrier as a part of the pathogenesis resulted from AlCl_3_ exposure (48). Nano particles are being designed to mimic LDL and interact with the LDL receptor, consequently triggering uptake by brain endothelial cells (49).

In other field of nano-HAp, Chu *et al*., (26) reported that nano-HAp abolished the apoptosis in breast tumor brain exposed to irradiation noxious. The bio-distribution of these particles is dependent on the characteristics of blood capillaries in the organs and tissues as well as the administration site, particle size, and particle surface properties. In this aspect, Xie (18) investigated the quantitative tissue distribution of intravenous nano-HAp in rats using^125^I radiolabeling and reported that nano-HAp was mainly accumulated in the soft tissues.

Histological analyses revealed that AlCl_3_ mediates progressive alterations in the brain tissue including pyknosis as well as vacuolization and atrophy in addition to inflammatory cell infiltration with neuronal degeneration and congestion in the blood vessels. These observations were very well corroborated with the previous reports (36, 50); it was found that AlCl_3_ causes histopathological lesions such as neuronal degeneration as cytoplasmic vacuolization hemorrhage, ghost cell, gliosis, and congestion in the blood vessels.

From dazzling of treatment with the nano-HAp, a complete recovery of the all histopathological torsion caused by administration of AlCl_3_ was reported. The intravenous injection of the nano-HAp not only diminished the sings of hazardous effect associated with AlCl_3_ administration but it restored all the cells structure. The histopathological examination revealed normal structure of all brain parts for the nano-HAp treated group.

## Conclusion

The work provided a newly interesting role of our nominated nano-HApas ideal biomedical for future clinical applications to restrain the brain injury due to the aluminum chloride over dose. With the rapid technical development in the nanometer-scaled particles in the medical field,it should be notified that the particles of the very small size have remarkable reactivity and detectable which must lead to concerns regarding the unknown risks of such materials. Therefore, prior to clinical applications, any toxicity side effects owing to the nano phase materials applications should be avoided.Further investigations in this field could provide valuable information and may have a great potential to revolutionize the field of brain damage attributed to heavy metals pollution.
